# Supplementary material for: CHCHD4 confers metabolic vulnerabilities to tumour cells through its control of the mitochondrial respiratory chain
Source: Cancer Metab. 2019 Mar 6;7:2. doi: 10.1186/s40170-019-0194-y (PMC6404347; doi:10.1186/s40170-019-0194-y)
Supplement: Supplementary file 1 — CHCHD4 siRNA and CHCHD4 shRNA sequences. (PDF 86 kb) [file 40170_2019_194_MOESM1_ESM.pdf]

siRNA/shRNA	**Sequence**
***CHCHD4* siRNA(1) target sequence**	GAGGAAACGUUGUGAAUUA
***CHCHD4* siRNA(2) target sequence**	AAGAUUUGGACCCUUCCAUUC
***CHCHD4* shRNA1 target sequence**	UGUUCCUUGUUAUUCCGAA
***CHCHD4* shRNA2 target sequence**	GGAUCGAAUCAUAUUUGUA

Additional file 1: CHCHD4 siRNA and CHCHD4 shRNA sequences. Table shows siRNA and shRNA sequences used for transient and stable knockdown of *CHCHD4* in cells.
