# Supplementary material for: CHCHD4 confers metabolic vulnerabilities to tumour cells through its control of the mitochondrial respiratory chain
Source: Cancer Metab. 2019 Mar 6;7:2. doi: 10.1186/s40170-019-0194-y (PMC6404347; doi:10.1186/s40170-019-0194-y)
Supplement: Supplementary file 2 — Q-PCR primer sequences. (PDF 141 kb) [file 40170_2019_194_MOESM2_ESM.pdf]

Gene	Forward Primer (5'-3')	Reverse Primer (5'-3')
CHCHD4	GAGCTGAGGAAGGGAAGGAT	AATCCATGCTCCTCGTATGG
GFER	GAGGAGTGTGCTGAAGACCT	CAGCTTGCGGTTCACTTCAT
AIF	GGCAAAATCGATAATTCTGTGGTTAGTC	CCACCAATTAGCAGGAAAGGAA
MT-ND1	GCCCCAACGTTGTAGGCCCC	AGCTAAGGTCGGGGCGGTGA
B2M	GAATGAGCGCCCGGTGTCCC	CCAAGCCAGCGACGCAGTG

Additional file 2: Q-PCR primer sequences. Table shows independent forward and reverse primers used for the Q-PCR analysis of the genes indicated.
