# Supplementary material for: CHCHD4 confers metabolic vulnerabilities to tumour cells through its control of the mitochondrial respiratory chain
Source: Cancer Metab. 2019 Mar 6;7:2. doi: 10.1186/s40170-019-0194-y (PMC6404347; doi:10.1186/s40170-019-0194-y)
Supplement: Supplementary file 5 — CHCHD4-mediated changes in the expression of nuclear-encoded CI subunits identified using SILAC analysis. (PDF 231 kb) [file 40170_2019_194_MOESM5_ESM.pdf]

Subunit	Total Residues	Cysteine Residues	Mean Fold Change
NDUFA2	172	8	1.18
NDUFA3	137	4	1.08
NDUFA5	172	5	1.11
NDUFA6	106	4	0.80
NDUFA7	99	2	1.13
NDUFA8*	84	0	1.17
NDUFA9	116	1	1.07
NDUFA10	154	2	1.17
NDUFA11	113	1	1.12
NDUFA12	377	3	1.11
NDUFA13	355	6	1.47
NDUFAF2	228	8	0.87
NDUFAF3	145	0	n.d.
NDUFAF4	144	0	0.95
NDUFB3	169	0	1.06
NDUFB4	184	4	1.33
NDUFB5	175	1	0.82
NDUFB6	98	0	0.96
NDUFB7*	129	1	1.12
NDUFB8	189	0	1.03
NDUFB9	128	0	1.04
NDUFB10*	172	3	1.13
NDUFB11	179	4	0.90
NDUFC2	153	1	1.36
NDUFS1	119	1	1.22
NDUFS2	727	18	1.14
NDUFS3	463	7	1.25
NDUFS4	264	2	1.22
NDUFS5*	175	0	0.89
NDUFS6	124	6	0.94
NDUFS7	206	4	1.08
NDUFS8	210	9	1.05
NDUFV1	464	12	1.21
NDUFV2	249	5	1.00

Additional file 5: CHCHD4-mediated changes in the expression of nuclear encoded CI subunits identified using SILAC analysis. Table shows mean fold change in the expression of CI subunits in response to elevated CHCHD4 expression. Data were calculated from two parallel labelling analyses (WT(H) vs Ctrl(L) and WT(L) vs Ctrl(H)) from 3 independent SILAC experiments. Total number of residues (amino acids) for proteins including the number of cysteine residues, and known CHCHD4 substrates (*) are indicated.
