# Supplementary material for: CHCHD4 confers metabolic vulnerabilities to tumour cells through its control of the mitochondrial respiratory chain
Source: Cancer Metab. 2019 Mar 6;7:2. doi: 10.1186/s40170-019-0194-y (PMC6404347; doi:10.1186/s40170-019-0194-y)
Supplement: Supplementary file 6 — CHCHD4 expression confers increased tumour cell sensitivity to CI inhibitors. (PDF 259 kb) [file 40170_2019_194_MOESM6_ESM.pdf]

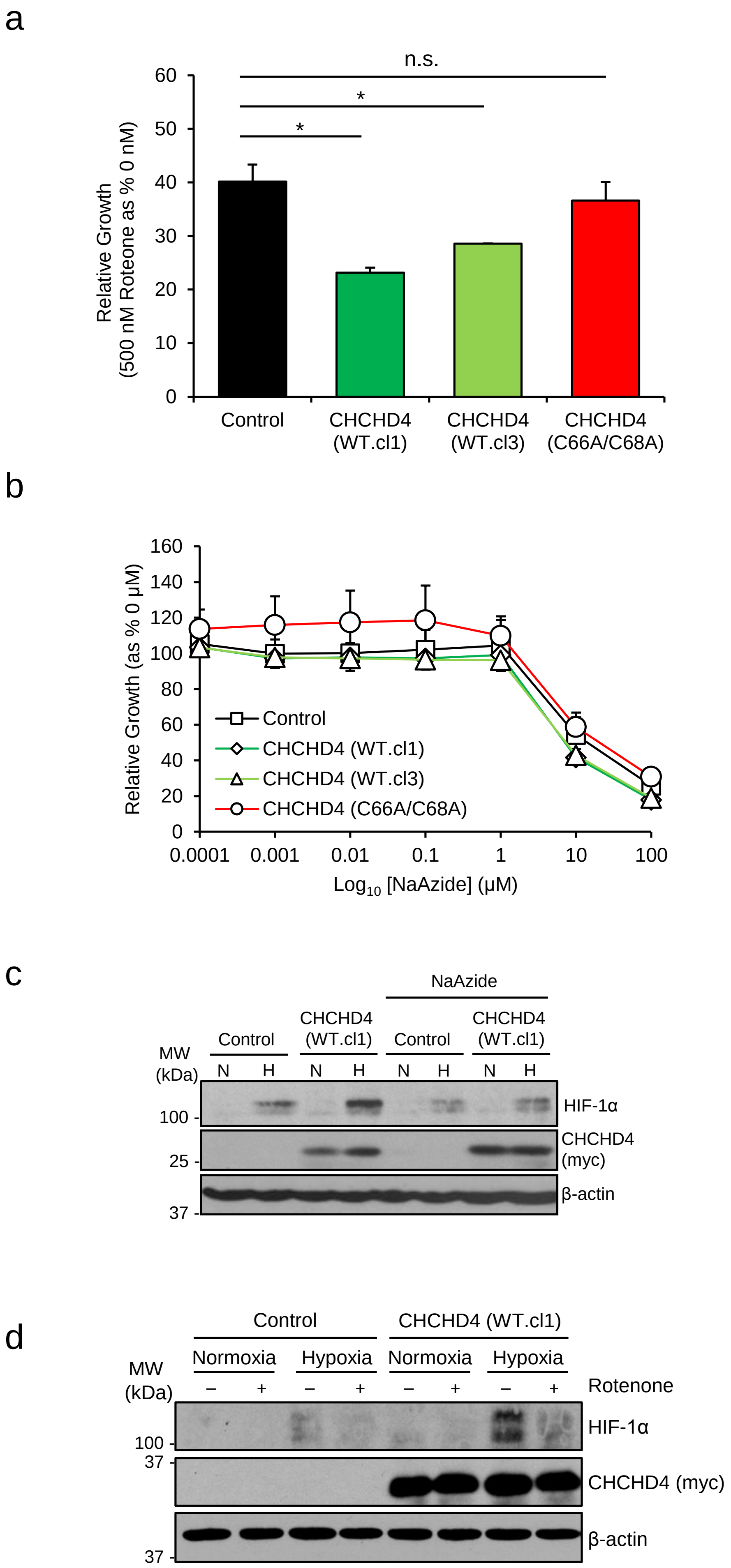

Additional file 6.

Additional file 6: CHCHD4 confers increased tumour cell sensitivity to CI inhibitors. **a** Graph shows relative growth rate of control U2OS (control) cells, two independent CHCHD4 (WT)-expressing cell clones (WT.cl1, WT.cl3) and CHCHD4 (C66A/C68A)-expressing cells incubated for 72h in hypoxia (1% O_2), in the absence or presence of rotenone (500 nM). Total cell protein assessed by SRB assay was used as a measure of cell growth. Relative growth calculated for rotenone-treated relative to untreated. n=3; mean ± SD; n.s. = not significant, * = $p<0.05$. **b** Graph shows relative growth rate of control U2OS (control) cells, two independent CHCHD4 (WT)-expressing cell clones (WT.cl1, WT.cl3) and CHCHD4 (C66A/C68A)-expressing cells incubated for 72h in hypoxia (1% O_2), in the absence or presence of sodium azide using a 10-fold dilution series (top concentration, 100 µM). Total cell protein assessed by SRB assay was used as a measure of cell growth. Relative growth calculated for rotenone-treated relative to untreated. n=3; mean ±SD. **c-d** Western blots show HIF-1α and CHCHD4 protein levels in control U2OS and CHCHD4 (WT)-expressing (WT.cl1) cells, untreated (-) or treated (+) with sodium azide (5 µM) (c) or rotenone (500 nM) (d) for 16h in normoxia or hypoxia (1% O_2). β-actin used as load control.
