# Supplementary material for: CHCHD4 confers metabolic vulnerabilities to tumour cells through its control of the mitochondrial respiratory chain
Source: Cancer Metab. 2019 Mar 6;7:2. doi: 10.1186/s40170-019-0194-y (PMC6404347; doi:10.1186/s40170-019-0194-y)
Supplement: Supplementary file 8 — CHCHD4-mediated HIF-α protein induction is blocked by NSC-134754 without affecting the respiratory chain. (PDF 219 kb) [file 40170_2019_194_MOESM8_ESM.pdf]

a

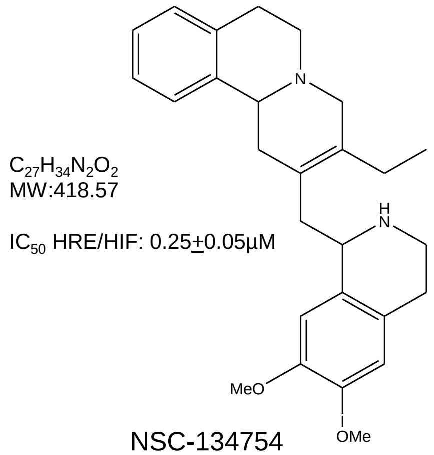

NSC-134754
(our revised structure)

b

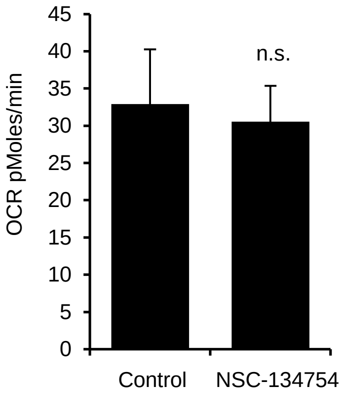

c

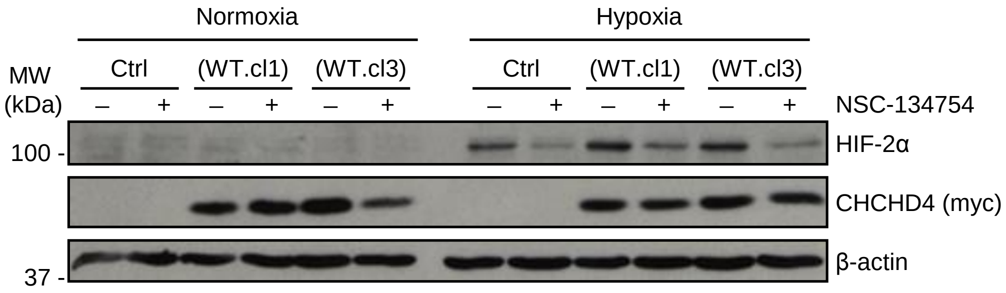

Additional file 8.

Additional file 8: CHCHD4-mediated HIF-α protein induction is blocked by NSC-134754 without affecting the respiratory chain. **a** Our revised chemical structure for NSC-134754. **b** Graph shows basal oxygen consumption rate (OCR, pMoles/min) in U2OS cells untreated or after treatment with NSC-134754 (10 μM) for 24h. n=3; mean ± SD; n.s. = not significant. **c** Western blots show HIF-2α and exogenous CHCHD4 (myc) protein levels in control U2OS and CHCHD4 (WT)-expressing cells (WT.cl1, WT.cl3), incubated in normoxia or hypoxia (1% O_2), untreated (NT) or treated with NSC-134754 (1 μM) for 8h. β-actin used as load control.
